# Estimated physical activity in Bavaria, Germany, and its implications for obesity risk: Results from the BVS-II Study

**DOI:** 10.1186/1479-5868-2-6

**Published:** 2005-06-08

**Authors:** Nina Schaller, Henrike Seiler, Stephanie Himmerich, Georg Karg, Kurt Gedrich, Günther Wolfram, Jakob Linseisen

**Affiliations:** 1 Human Nutrition and Cancer Prevention, Technical University of Munich, Alte Akademie 16, 85350 Freising-Weihenstephan, Germany; 2 Consumer Economics, Technical University of Munich, Weihenstephaner Steig 17, 85350 Freising-Weihenstephan, Germany; 3 Department of Food and Nutrition, Technical University of Munich, Alte Akademie 16, 85350 Freising-Weihenstephan, Germany; 4 Division of Clinical Epidemiology, German Cancer Research Centre, Im Neuenheimer Feld 280, 69120 Heidelberg, Germany

## Abstract

**Background:**

Adequate physical activity (PA) is considered as a key factor in the fight against the obesity epidemic. Therefore, detailed description of the actual PA and its components in the population is necessary. Additionally, this study aims to investigate the association between PA and obesity risk in a representative population sample in Bavaria, Germany.

**Methods:**

Data from 893 participants (age 13–80 years) of the Bavarian Food Consumption Survey II (BVS II) were used. In each participant, three computer-based 24-hour recalls were conducted by telephone assessing type and duration of PA in the domains occupation, sports, other strenuous leisure time activities (of mostly moderate intensity) as well as TV/PC use in leisure time and duration of sleeping. After assigning metabolic equivalents (METs) to each activity, estimates of energy expenditure (MET*h) and total daily PA level (PAL_est._) were calculated. In a subgroup of adults (n = 568) with anthropometric measurements logistic regression models were used to quantify the impact of PA on obesity risk.

**Results:**

Estimated average PA in women and men was 38.5 ± 5.0 and 40.6 ± 9.3 MET*h/d, respectively, corresponding to PAL_est. _values of 1.66 ± 0.22 and 1.75 ± 0.40. Obese subjects showed lower energy expenditure in the categories sports, occupation, and sleeping, while the time spent with TV/PC during leisure time was highest. This is confirmed in logistic regression analyses revealing a statistically significant association between obesity and TV/PC use during leisure time, while sports activity was inversely related to obesity risk. Overall, less than 1/3 of the study participants reached the recommended PAL of ≥ 1.75. Subjects within the recommended range of PA had an about 60 % (odds ratio = 0.43; 95% CI: 0.21–0.85) reduced risk of obesity as compared to inactive subjects with a PAL_est. _<1.5.

**Conclusion:**

Based on the results of short-term PA patterns, a major part of the Bavarian adult population does not reach the recommendations (PAL>1.75; moderate PA of > 30 min/d). Despite the limitations of the study design, the existing associations between sports activity, TV/PC use and obesity risk in this population give further support to the recommendation of increasing sports activity and reducing sedentary behaviour in order to prevent rising rates of obesity.

## Background

Globally, there are more than 1 billion overweight adults, at least 300 million of them obese. These alarming facts published by the World Health Organisation (WHO) [1] demonstrate that obesity has reached epidemic dimension in developed as well as in developing countries. Consequences on health range from several non-fatal but debilitating disorders that reduce quality of life to increased risk of premature death because of serious chronic diseases. Besides genetic factors and food consumption patterns exceeding the individual energy need, a sedentary lifestyle with lack of physical activity (PA) is one of the key causes [2]. The relationship between obesity, PA and chronic diseases is close and several epidemiological studies could show that regular PA can prevent from obesity and related chronic diseases, such as type-2 diabetes, cardiovascular disease, hypertension, stroke, cancers of different sites, osteoporosis, and contribute to maintain mental health [1,3]. Thus, PA promotes health and well-being and has also enormous economic benefits considering the health care costs that could be attributed to obesity. However, the question of the adequate dose of exercise is still a matter of debate [4-6].

In order to provide a solid basis for obesity prevention strategies detailed knowledge of PA patterns in the target population is necessary. Therefore, we assessed short-term PA and sedentary behaviour of the Bavarian population by means of three unannounced 24-h recalls. Different activity domains contributing to total daily energy expenditure are described and their impact on obesity risk is quantified. Additionally, PA estimates in the Bavarian population are compared with current recommendations to prevent obesity and promote well-being and health.

## Methods

### Study Design

The Bavarian Nutrition Survey II (BVS II) is designed as a representative study of the Bavarian population to investigate dietary habits and PA. From September 2002 until June 2003, 1050 subjects aged 13–80 years were recruited by a three-stage random route sampling procedure from the German-speaking Bavarian population. This recruitment procedure included the selection of 42 communities as so-called sampling points (stratified by county and community characteristics), a random walk (every third household) with a given start address, and a random selection of one household member who meets the selection criteria. At baseline, subjects' characteristics, lifestyle, socio-economic and health status were assessed by means of a computerized face-to-face interview. Within the following two weeks, participants were contacted by telephone on two workdays and one weekend day for recalling their dietary intake as well as PA on the day before. Within six weeks after recruitment, all adult study subjects (=18 years) were invited to their nearest health office for blood sampling and standardized anthropometric measurements.

Participation rate in the whole study was 71 % (n = 1050). All adults that completed at least one 24-h dietary recall (n = 879) were invited to the health offices; from 65 % (n = 568) of those approached blood samples and anthropometric measurements could be obtained. For the present evaluation, 893 subjects who completed at least two 24-h activity-recalls were included. Within this group standardized anthropometric measurements were available from 552 subjects (61.8 %). All participants gave their written informed consent. The study was approved by the local ethical committee.

### Assessment of Physical Activity

According to a method described and validated by Matthews et al. [14], information on the short-term PA of each subject was collected by means of three unannounced computer-assisted telephone interviews. Trained interviewers asked the study participants to recall the exact type and time spent in activities of the following 5 categories during the last 24 hours: occupation, sports, other strenuous leisure time activities (LTPA_strenuous_), TV or PC use in leisure time and sleeping. In the categories sports and LTPA_strenuous_, the interviewers used a list of common activities laid on the screen in order to give examples to the participants and to fasten the interview process. Different types of walking (including walking for pleasure) were attributed to the category 'sports' since this type of activity is very important in older age; the category LTPA_strenuous _included mainly leisure time PAs of moderate and vigorous intensity, such as different types of gardening, homemaking and household activities, or child caring. Although the wording of the question ('strenuous') may imply vigorous activities only, we actually assessed mainly activities of moderate intensity by means of this question (see results).

Based on the results of their validation study, Matthews et al. [14] concluded that a series of three unannounced 24-h PA recalls provides an assessment of PA comparable to other short-term PA assessments that utilize activity monitors (Actillume monitoring) or the Baecke questionnaire. Deattenuated Pearson correlation coefficients between results from the 24-h recalls and the Baecke questionnaire ranged from 0.34–0.68 (p < 0.01). A correlation coefficient of 0.64 (p < 0.01) was reported for the association between 24-h recall results (total MET*h/d) and the Actillume measures (counts*min^-1 ^*d^-1^). They assessed four intensities of activity (light, moderate, vigorous, and very vigorous) in each of three activity domains (household, occupational, leisure-time) as well as sleeping time, and assigned 1.5 MET for light, 4.0 MET for moderate, and 6.0 MET for vigorous activities [14]. In our study, we more precisely assessed the time and type of activity spent in different PA categories and assigned individual MET values; however, except for TV/PC use, we did not actively assess the time spent with light activities during leisure time.

As described in the compendium of physical activities by Ainsworth et al. [7,8], multiples of the metabolic equivalent (METs) were used to estimate the relative intensity of each reported activity with one MET equal to the standard for resting energy expenditure (roughly 3.5 ml of oxygen consumed per kilogram of body weight/min) for the average adult. According to the assigned MET-values, all self-reported activities were classified as light (< 3 METs), moderate (3–6 METs) or vigorous (>6 METs) [4,8].

The MET-values of occupational activities were determined by a combination of self-reported work-intensity (ranging from mainly sitting to laborious physical workload or actually not working) and respective job-title. When a description of activities was missing or the provided information unclear standardized mean MET-values were assigned. In particular, if job activities of students and retired persons were reported that could not be classified, a MET-value of 1.85 representing light work was assumed to be applicable. Type and intensity of the activity of homemakers was also difficult to evaluate; only for this group all reported strenuous activities belonging to the area of household activities were considered as being included in occupational household work and, therefore, not attributed to LTPA_strenuous_. To acknowledge homemakers' activities as full occupation, we filled up the reported working time to at least 8 hours of work per weekday for all homemakers under 65 years. An intensity level of 2.5 METs representing "multiple household tasks all at once, light effort" [8] was assigned.

Energy expenditure estimates (MET*h) independent from body weight were calculated by multiplying the reported duration of any activity (h) by respective intensity (MET) [7,8]. By summing up all activities, participants' daily MET*h were obtained for the different activity domains, e.g. sports-MET*h per day. In order to estimate a total daily PA score, it was necessary to introduce a new activity domain, called non-reported PA during leisure time (LTPA_non-reported_), according to a method described by Norman et al. [9,10]. The difference between 24 hours per day and the total duration of self-reported activity/inactivity was considered as LTPA_non-reported_. These unknown activities were multiplied by an estimated MET-value of 1.75, which is between the suggested values of 1.5 MET [14] and 2.0 MET [9,10]. The intensity factor corresponds to the mean of sitting (1.5 MET) and light home and self-care activities (2.0 MET) [7,8]. Since our study participants mentioned also several light activities under the category LTPA_strenuous _– which were multiplied with the most exact MET value given by Aintsworth et al. – we tried not to overestimate the remaining non-reported time.

The single recalls were weighted for weekday or weekend day to calculate a subject's total daily short-term PA and its components. We also estimated the participants' short-term PA level (PAL_est._) by dividing the individual total daily PA score (MET*h/d = kcal/(kg body weight*d) =~ 1 kcal/(kg b.w.*min) [7,8]) by the minimum score of 23.2 MET*h/d (assumption of 8 hours of sleep × 0.9 MET and 16 h being awake, but resting × 1.0 MET) [11]. Since 23.2 MET*h should reflect resting metabolic rate (RMR) expressed in units of MET*h, the resulting ratio gives the multiple of RMR [11], similar to the PAL value. However, it has to be emphasized that the calculated PAL_est. _values are of limited precision as compared to the PAL values mainly derived by means of the doubly labelled water method.

### Case definition

To assess the prevalence of overweight and obesity, the subjects' body mass index (BMI) was calculated as measured weight divided by the square of measured height (kg/m^2^). Self-reported figures were used for subjects who did not undergo anthropometric measurements. Following the WHO-guidelines [12] participants were classified into six categories as being underweight (<18.5 kg/m^2^), normalweight (18.5-<25 kg/m^2^), overweight (25-<30 kg/m^2^), obese grade I (30-<35 kg/m^2^), obese grade II (35-<40 kg/m^2^) and obese grade III (≥ 40 kg/m^2^). All obese subjects (n = 144) with BMI ≥ 30 kg/m^2 ^were considered as cases and all other study participants served as controls in the logistic regression analyses.

### Statistical Analysis

The given descriptive results were weighted to correct for the deviation of the study group from the distribution of gender, age, and living area in the underlying Bavarian population. Since the PA data were not normally distributed, median and interquartile range are presented. Comparisons between gender and BMI groups were made by means of the Mann-Whitney U test. In order to examine the association between PA and obesity risk, logistic regression models were used. Risk calculations were conducted only for the subgroup with standardized measurement of weight and height. Additionally, subjects with an energy intake below 80% of the estimated basal metabolic rate (BMR, calculated by WHO-equations [13]) were excluded from risk estimations because of an increased likelihood of misreporting of PA. Thus, risk evaluation was conducted in a subgroup of 507 subjects. The activity estimates (MET*h/d) over each activity-domain as well as the total daily activity (MET*h/d and PAL_est_, respectively) were divided into four groups according to the distribution in the entire study population or by predefined cut points. Odds ratios (OR) and corresponding 95% confidence intervals (CI) are given for models adjusted for sex, age (< 18 y, 18-<30 y, 30-<40 y, 40-<50 y, 50-<65 y, ≥ 65 y), energy intake (kcal/100/d), smoking (never, former, current) and socio-economic status (low, low-medium, medium, medium-high, high). Categorization of socio-economic status is based on the value of three characteristics on a point-scale including household net income, educational level of the one who is being interviewed and career position of the principal earner. Tests on trend were calculated using the quartile-based PA scores as a continuous variable as well as using the continuous variables (in MET*h/d). All statistical analyses were performed by means of the SPSS 11.0 software package (SPSS Inc., Chicago, USA).

## Results

### Baseline characteristics and prevalence of obesity

Baseline characteristics of the study participants are summarized in Table 1. Significant gender differences existed for BMI groups, socioeconomic status, employment level, smoking habits and marital status; also anthropometric measures as well as basal metabolic rate (BMR) and energy intake differed by gender. The proportion of obese subjects in the whole sample (n = 893) was estimated to 17.1% in women and 16.1% in men. Excluding subjects with self-reported weight and height, the prevalence of obesity was even higher with 19.6% in women and 20.4% in men (overall 20.0%).

**Table 1:** Baseline characteristics of the study participants^1^.

	**Total (n = 893)**		**Women (n = 528)**		**Men (n = 365)**		**p-value**^2^
				
	**n**	**%**	**n**	**%**	**n**	**%**	
**Age (years)**							0.713
<18	48	7.4	20	6.4	28	8.6	
18-<30	99	13.5	65	14.0	34	12.9	
30-<40	196	21.0	125	20.2	71	21.8	
40-<50	182	19.0	119	19.7	63	18.2	
50-<65	228	23.8	129	23.5	99	24.2	
≥65	140	15.2	70	16.2	70	14.1	
**Body mass index (kg/m^2^)**							<0.001
underweight (<18.5)	35	4.2	25	5.9	10	2.4	
normal (18.5-<25)	402	44.9	265	49.3	137	40.0	
overweight (25-<30)	312	34.1	154	27.4	158	41.5	
obese (≥30)	144	16.6	84	17.1	60	16.1	
grade I (30-<35)	99	11.2	54	10.5	45	12.0	
grade II (35-<40)	31	3.6	17	3.3	14	3.8	
grade III (≥40)	14	1.9	13	3.5	1	0.2	
**Socioeconomic status**							0.001
low	133	13.6	81	14.3	52	12.9	
low-medium	230	25.5	129	25.7	101	25.4	
medium	262	29.3	163	29.9	99	28.7	
medium-high	178	21.2	118	24.0	60	18.2	
high	90	10.3	37	6.2	53	14.8	
**Employment**							<0.001
employed	429	48.1	241	41.2	188	55.6	
homemaker	152	13.9	151	26.5	1	0.2	
student/articled	78	12.3	35	10.3	43	14.6	
unemployed/other	36	4.1	14	2.2	22	6.2	
retired	198	21.5	87	19.7	111	23.4	
**Smoking status**							<0.001
never	473	52.3	320	61.2	153	42.6	
former	183	21.0	83	16.0	100	26.3	
current	236	26.7	124	22.6	112	31.1	
missing Data	1	0.1	1	0.2	0	0.0	
**Marital status**							<0.001
single	176	21.6	82	17.1	94	26.6	
married/cohabiting	578	67.0	337	65.8	241	68.4	
divorced/widowed	138	11.3	108	17.1	30	5.0	
missing data	1	0.0	1	0.0	0	0.0	

	**mean ± SD**						
**Height (cm)**	169.6 ± 9.1		164.0 ± 6.7		175.5 ± 7.3		<0.001
**Weight (kg)**	74.3 ± 15.6		68.1 ± 13.9		81.0 ± 14.6		<0.001
**BMI (kg/m^2^)**	25.8 ± 5.2		25.5 ± 5.8		26.2 ± 4.3		0.038
**BMR (kcal)**	1601 ± 253		1419 ± 132		1801 ± 197		<0.001
**Energy intake (kcal)^3^**	2001 ± 667		1704 ± 529		2326 ± 652		<0.001

^1^weighted for deviation from the underlying Bavarian population (sex, age, region)

^2^Chi-Square test or independent-samples t-test for gender differences

^3^3 × 24-hour dietary recall

### Estimated Physical Activity

Estimates of PA by activity domain (MET*h/d) and intensity are given in Table 2, including also the corresponding duration of activities (h/d). Men as compared to women showed significantly higher values in total scores of sports activity, TV/PC use and total daily activity, while women reported a significantly longer sleeping time per day. This is also reflected in results by intensity sub-groups with men spending more time in PA with moderate or vigorous intensity. The most important intensity subgroup was occupational PA of light intensity showing the highest mean energy expenditure for both men and women. Non-reported time of PA in the 24-hour recalls was higher in women than in men. Total daily PA was estimated to 37.35 (5.58) MET*h/d (median, interquartile range) in women and 37.92 (8.80) in men, corresponding to PAL_est. _values of 1.61 (0.24) and 1.63 (0.38), respectively.

**Table 2:** Estimated physical activity (h/d and MET*h/d) by sex, type, and intensity of activity^1^.

Type and intensity* of activity/inactivity	**Total (n = 893)**		**Women (n = 528)**		**Men (n = 365)**		p-value^2 ^(MET*h/d)
	**h/d**	MET*h/d	**h/d**	MET*h/d	**h/d**	MET*h/d	
		
	Median (Interquatile range)						
Occupation							
total	2.86 (5.71)	5.36 (14.00)	2.86 (5.71)	5.14 (14.29)	2.95 (6.07)	6.83 (12.60)	0.799
*light*	*82.6%*^#^	*68.1%*	*89.9%*	*82.4%*	*75.8%*	*55.2%*	
*moderate*	*13.2%*	*21.5%*	*8.8%*	*13.6%*	*17.7%*	*28.7%*	
*vigorous*	*4.0%*	*10.4%*	*1.7%*	*4.0%*	*6.5%*	*16.2%*	
Sports							
total	0.12 (0.63)	0.48 (3.34)	0.08 (0.53)	0.38 (2.83)	0.14 (0.73)	0.59 (4.50)	0.028
*moderate*	*57.5%*	*43.1%*	*66.7%*	*52.9%*	*51.1%*	*36.4%*	
*vigorous*	*40.0%*	*56.9%*	*33.3%*	*46.6%*	*48.9%*	*63.6%*	
LTPA_strenuous_^3^							
total	0.00 (0.54)	0.00 (1.71)	0.00 (0.57)	0.00 (2.10)	0.00 (0.36)	0.00 (1.55)	0.893
*light*	*5.0%*	*3.2%*	*5.4%*	*4.4%*	*4.8%*	*2.2%*	
*moderate*	*92.5%*	*92.4%*	*91.95*	*90.4%*	*95.2%*	*94.0%*	
*vigorous*	*2.5%*	*4.5%*	*2.7%*	*5.2%*	*2.4%*	*3.8%*	
							
TV/PC_leisure time_^4^	1.64 (1.82)	1.64 (1.82)	1.38 (1.52)	1.38 (1.52)	2.00 (1.96)	2.00 (1.96)	<0.001
Sleeping^5^	7.43 (1.39)	6.69 (1.25)	7.56 (1.37)	6.80 (1.23)	7.31 (1.36)	6.58 (1.22)	0.016
LTPA_non-reported_^6^	10.93 (4.04)	19.12 (7.08)	11.26 (4.17)	19.71 (7.30)	10.61 (4.08)	18.56 (7.14)	<0.001
							
Total daily activity score							
MET*h		37.52 (7.18)		37.35 (5.58)		37.92 (8.80)	<0.001
PAL_est._		1.62 (0.31)		1.61 (0.24)		1.63 (0.38)	<0.001

^1 ^Weighted for deviation from the underlying Bavarian population (sex, age, region)

^2 ^Mann-Whitney U-test

^3 ^LTPA_strenuous _= strenuous leisure time physical activity

^4 ^TV/PC_leisure time _(1.0 MET)

^5 ^Sleeping (0.9 MET)

^6 ^LTPA_non-reported _= non-reported leisure time physical activity (1.75 METs)

* Light (<3 METs), moderate (3–6 METs), vigorous (>6 METs)

^# ^Percentage of mean h/d and mean MET*h/d, respectively, of the corresponding activity domain

Table 3 shows the results for the estimated PA by type and intensity level in different BMI categories. Obese subjects reported less participation in occupational (women) and sports (men) activities but performed more LTPA_strenuous _than non-obese women and men. On the contrary, the time spent with TV/PC use during leisure time was highest in overweight and obese subjects. Sleeping time was shortest among obese women while underweight subjects slept most. Total daily activity scores were lowest in obese and underweight subjects, thus, the difference between obese and non-obese subjects did not reach statistical significance.

**Table 3:** Estimated physical activity (MET*h/d) by weight class (BMI), type and, intensity of activity.

	**Underweight (<18.5 kg/m^2^)**	**Normalweight (18.5-<25)**	**Overweight (25-<30)**	**Obese (≥30)**	**p-value**
Type and intensity* of activity/inactivity	**Women (n=528)**				**difference^2 ^obese vs. others)**
	**n = 25**	**n = 265**	**n = 154**	**n = 84**	
	Median (Interquatile range)				
Occupation					
total	2.93 (9.76)	5.79 (14.29)	5.35 (14.29)	2.41 (11.68)	0.033
*light*	*55.8%*^#^	*83.8%*	*81.6%*	*89.7%*	
*moderate*	*18.8%*	*12.2%*	*16.8%*	*10.3%*	
*vigorous*	*25.3%*	*3.9%*	*1.8%*	*0%*	
Sports					
total	1.62 (3.24)	0.63 (3.12)	0.00 (2.30)	0.00 (1.73)	0.105
*moderate*	*39.9%*	*47.0%*	*54.8%*	*80.4%*	
*vigorous*	*60.1%*	*53.0%*	*43.9%*	*19.6%*	
LTPA_strenuous_^3^					
total	0.00 (0.00)	0.00 (1.61)	0.00 (3.21)	0.00 (2.41)	0.026
light	*0.0%*	*5.3%*	*2.2%*	*5.4%*	
moderate	*100.0%*	*87.6%*	*89.9%*	*94.6%*	
vigorous	*0 %*	*7.1%*	*7.3%*	*0%*	
					
TV/PC_leisure time_^4^	1.21 (1.11)	1.11 (1.33)	1.69 (2.04)	1.78 (1.61)	0.004
					
Sleeping^5^	7.90 (2.07)	6.80 (1.24)	6.75 (1.03)	6.75 (1.25)	0.332
					
LTPA_non-reported_^6^	20.77 (8.20)	19.26 (7.72)	19.49 (7.45)	19.89 (6.66)	0.325
					
Total daily activity score					
MET*h/d	36.56 (7.08)	37.78 (6.00)	37.20 (5.97)	36.70 (4.95)	0.083
PAL_est._	1.58 (0.31)	1.63 (0.26)	1.60 (0.26)	1.58 (0.21)	0.083

	**Men (n = 365)**				
	**n = 10**	**n = 137**	**n = 158**	**n = 60**	

	Median (Interquatile range)				
Occupation					
total	3.47 (6.51)	7.48 (13.68)	5.09 (12.45)	3.01 (12.28)	0.098
*light*	*100.0%*	*60.6%*	*46.0%*	*61.4%*	
*moderate*	*0%*	*18.2%*	*46.5%*	*10.7%*	
*vigorous*	*0%*	*21.3%*	*7.5%*	*28.1%*	
Sports					
total	7.52 (15.67)	1.29 (7.86)	0.39 (3.21)	0.00 (2.05)	0.006
*moderate*	*13.2%*	*33.0%*	*48.5%*	*28.6%*	
*vigorous*	*86.9%*	*67.0%*	*51.0%*	*71.4%*	
LTPA_strenuous_^3^					
total	0.00 (0.00)	0.00 (0.54)	0.00 (2.86)	0.29 (4.39)	0.007
*light*	*0%*	*1.9%*	*3.1%*	*0.7%*	
*moderate*	*100.0%*	*87.5%*	*94.6%*	*98.7%*	
*vigorous*	*0%*	*11.5%*	*2.2%*	*0.7%*	
					
TV/PC_leisure time_^4^	1.46 (2.53)	1.77 (1.62)	2.07 (1.97)	2.46 (1.92)	0.001
					
Sleeping^5^	8.21 (1.63)	6.62 (1.13)	6.56 (1.28)	6.48 (0.90)	0.040
					
LTPA_non-reported_^6^	15.80 (6.43)	18.10 (7.45)	18.66 (6.95)	19.50 (7.84)	0.413
					
Total daily activity score					
MET*h/d	37.54 (11.56)	38.94 (8.81)	37.19 (8.42)	37.42 (8.92)	0.087
PAL_est._	1.62 (0.50)	1.68 (0.38)	1.60 (0.36)	1.61 (0.38)	0.087

^1 ^Weighted for deviation from the underlying Bavarian population (sex, age, region)

^2 ^Mann-Whitney U-test

^3 ^LTPA_strenuous _= strenuous leisure time physical activity

^4 ^TV/PC_leisure time _(1.0 MET)

^5 ^Sleeping (0.9 MET)

^6 ^LTPA_non-reported _= non-reported leisure time physical activity (1.75 METs),

* Light (<3 METs), moderate (3–6 METs), vigorous (>6 METs)

^# ^Percentage of mean MET*h/d of the corresponding activity domain

### Physical Activity and Risk of Obesity

Risk estimations in the subgroup with measured weight and height and after exclusion of suspected miss-reporters revealed a significant inverse association between obesity and sports activity (Table 4). After adjusting for sex, age, energy intake, socio-economic and smoking status the odds ratio (CI) for the subjects with more than 5 MET*h/d of sports activities was 0.37 (0.16–0.85; p = 0.037 for trend_cont._) as compared to subjects with no sports activity. The use of TV/PC in leisure time was positively associated with obesity. As compared to subjects with less than 1 MET*h/d (1^st ^quartile), the ORs (95% CI) in the 2^nd^, 3^rd^, and 4^th ^quartiles, were 3.12 (1.42–6.87), 2.92 (1.29–6.58), and 2.51 (1.07–5.87), respectively (p = 0.059 for trend_cont._). Obesity risk tends to decrease with increasing sleeping (p = 0.062 for trend_cont._), except for the small group with > 8 MET*h/d spent with sleeping.

**Table 4:** Obesity risk by types of physical activity and total physical activity (n = 507^#^)*.

		**Quartiles**					
**Type of activity**		**Q1**	**Q2**	**Q3**	**Q4**	**p**_trend(cat.)_^1^	**p**_trend(cont.)_^2^
Occupation							
	No. cases/controls	42 / 146	17 / 87	15 / 106	15 / 79		
	Limits of quartiles (MET*h/d)	0.00	0.25-<8.00	8.00-<14.50	≥14.50		
	Median (MET*h/d)	0.00	4.29	11.23	17.68		
	odds ratio (95% Cl)	0.60 (0.28–1.30)	1 (ref.)	0.83 (0.38–1.83)	0.97 (0.44–2.18)		
Sports							
	No. cases/controls	47 / 185	21 / 75	13 / 84	8 / 74		
	Limits of quartiles (MET*h/d)	0.00	0.10-<2.00	2.00-<5.00	≥5.00		
	Median (MET*h/d)	0.00	0.94	2.86	8.57		
	odds ratio (95% Cl)	1 (ref.)	0.91 (0.49–1.69)	0.69 (0.34–1.39)	0.37 (0.16–0.85)	0.017	0.037
LTPA_strenuous_^3^							
	No. cases/controls	52 / 247	12 / 61	10 / 49	15 / 61		
	Limits of quartiles (MET*h/d)	0.00	0.10-<2.00	2.00-<4.50	≥4.50		
	Median (MET*h/d)	0.00	1.07	3.21	6.93		
	odds ratio (95% Cl)	1 (ref.)	0.94 (0.45–1.94)	0.88 (0.40–1.93)	0.74 (0.37–1.48)	0.393	0.650
TV/PC_leisuretime_							
	No. cases/controls	10 / 129	29 / 116	26 / 93	24 / 80		
	Limits of quartiles (MET*h/d)	<1.00	1.00-<2.00	2.00-<3.00	≥3.00		
	Median (MET*h/d)	0.5	1.43	2.34	3.65		
	odds ratio (95% Cl)	1 (ref.)	3.12 (1.42–6.87)	2.92 (1.29–6.59)	2.51 (1.07–5.89)	0.081	0.059
Sleeping							
	No. cases/controls	27 / 97	36 / 166	21 / 135	5 / 20		
	Limits of quartiles (MET*h/d)	<6.00	6.00-<7.00	7.00-<8.00	≥8.00		
	Median (MET*h/d)	5.46	6.57	7.35	8.23		
	odds ratio (95% Cl)	1 (ref.)	0.76 (0.42–1.37)	0.55 (0.28–1.07)	1.08 (0.33–3.51)	0.217	0.062
LTPA_non-reported_^4^							
	No. cases/controls	15 / 96	22 / 104	27 / 127	25 / 91		
	Limits of quartiles (MET*h/d)	<15.00	15.00-<19.00	19.00-<23.00	≥23.00		
	Median (MET*h/d)	13.25	16.94	20.89	24.98		
	odds ratio (95% Cl)	1 (ref.)	1.25 (0.59–2.64)	0.75 (0.35–1.60)	0.94 (0.43–2.04)	0.587	0.275
Total daily PA score (PAL_est._)							
	No. cases/controls	31 / 92	39 / 189	10 / 87	9 / 50		
	Limits of quartiles (PAL_est._)	<1.5	1.5-<1.75	1.75-<2.00	>≥2.00		
	Median (PAL_est._)	1.45	1.6	1.83	2.15		
	odds ratio (95% Cl)	1 (ref.)	0.59 (0.33–1.05)	0.35 (0.16–0.81)	0.56 (0.23–1.37)	0.038	0.728

^#^subgroup with measured weight and height and exclusion of suspected miss-reporters

*adjusted for sex, age, energy intake (kcal/100/d), socioeconomic status (low, medium-low, medium, medium-high, high) and smoking status (never, former, current)

^1^tests on trend by using quartile-based PA scores as a continuous variable [p_trend(cat.)_]

^2^tests on trend by using uncategorized PA scores (MET*h) as a continuous variable [p_trend(cat.)_]

^3^LTPA_strenuous _= strenuous leisure time physical activity

^4^LTPA_non-reported _= non-reported leisure time physical activity

Obesity was inversely associated with total daily PA (PAL_est _values). The risk estimates declined over increasing PA quartiles (except for the 4th quartile) reaching statistical significance for the 3rd quartile with PAL values between 1.75 and 2.0. Combining all subjects with a PAL value of 1.75 or higher in one category (Q3 + Q4) the OR (95% CI) was 0.43 (0.21–0.85) indicating a strong inverse association with obesity.

### Meeting of Physical Activity Recommendations

When comparing the calculated PAL_est. _values in our population with the WHO recommendation of (measured) PAL =1.75, only 26.8% of women and 36.4% of men met this recommendation. The rates declined with increasing BMI and age (Table 5), noting some exceptions (underweight subjects, age-groups < 18 and 40-<50). The public health recommendation of at least 30 minutes of moderate PA per day was met by 53.5% of women and 58.6% of men, including moderate to vigorous activities (≥ 3 METs) out of all relevant PA categories (occupation, sports, LTPA_strenuous_). Only the proportion of subjects with at least moderate (≥ 3 METs) sports activity for 30 min/d or longer was identified to decline with increasing BMI category; no such association can be seen when considering all leisure time PA or total PA (including also occupational activities). This indicates that a public health recommendation for obesity prevention in terms of an overall PA of at least 30 min/d of higher than light intensity may not work in this population. Such recommendations should be focused on sport activities only, a category that includes also walking.

**Table 5:** Participants^1 ^meeting physical activity recommendations.

**a) by BMI (kg/m^2^)**	** Total ** **(n = 893)**		**Underweight** **(BMI <18.5)** **(n = 35)**		** Normalweight ** **(BMI 18.5-<25)** **(n = 402)**		** Overweight ** **(BMI 25-<30)** **(n = 312)**		** Obese ** **(BMI ≥ 30)** **(n = 144)**			** **	** **	
					
	**n**	**%**	**n**	**%**	**n**	**%**	**n**	**%**	**n**	**%**				
				
**WHO-recommendation: PAL ≥ 1.75^§^** **Public health recommendation (ACSM/CDC): ≥ 30 min/d of moderate-intense (≥ 3 METs) activity**	275	31.4	13	29.7	137	35.1	93	30.8	32	22.8				
in all activity-domains	498	55.9	20	48.6	214	53.7	183	59.9	81	55.9				
in leisure time (sports, LTPA_strenuous_^2^)	452	50.1	17	40.5	199	49.2	162	52.2	74	51.0				
in sports only	266	30.2	17	40.5	135	35.2	83	26.8	31	20.7				
**b) by age (years)**	**Total** **(n = 893)**		**<18** **(n = 48)**		**18-<30** **(n = 99)**		**30-<40** **(n = 196)**		**40-<50** **(n = 182)**		**50-<65** **(n = 228)**		**≥65** **(n = 140)**	

	**n**	**%**	**n**	**%**	**n**	**%**	**n**	**%**	**n**	**%**	**n**	**%**	**n**	**%**

**WHO-recommendation: PAL ≥ 1.75^§^** **Public health recommendation (ACSM/CDC): ≥ 30 min/d of moderate-intense (≥ 3 METs) activity**	275	31.4	15	34.8	42	41.5	61	31.3	75	42.2	67	30.3	15	9.0
in all activity domains	498	55.9	33	67.7	54	52.5	96	49.2	105	57.8	134	59.1	76	55.6
in leisure time (sports, LTPA_strenuous_^2^)	452	50.2	33	67.7	52	50.0	78	37.7	93	49.7	122	53.8	74	54.1
in sports only	266	30.1	32	63.6	35	35.6	44	22.0	54	28.7	63	28.8	38	23.9

^1^weighted for deviation from the underlying Bavarian population; ^2^LTPA_strenuous _= strenuous leisure time physical activity

^§ ^comparing the PAL_est. _values from the present study with limited accuracy with a recommendation based on precise PAL values given by the WHO

## Discussion

**Figure 1:**
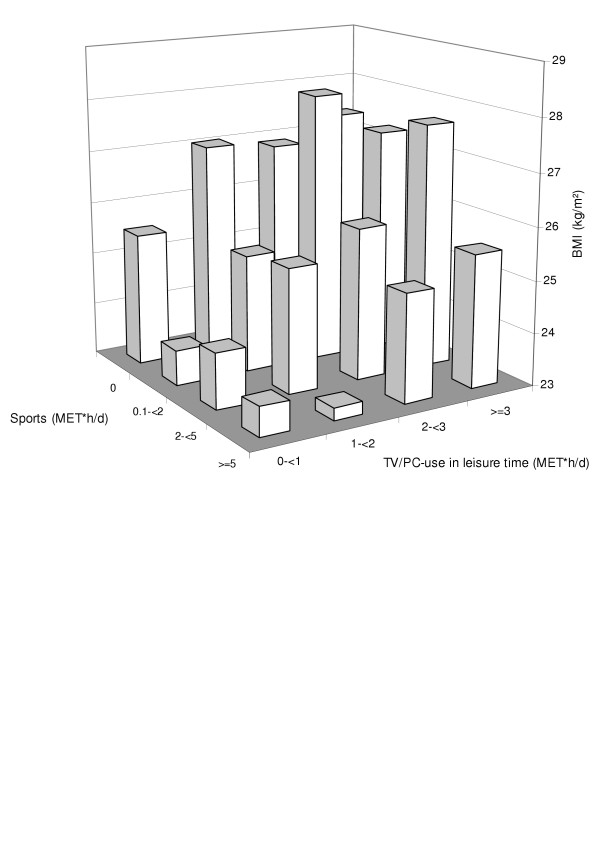
Mean BMI (kg/m^2^) by sports activity and use of TV/PC_leisure time _(MET*h/d; n = 893)

The results of our investigation revealed that higher PA in the category sports and less use of TV/PC during leisure time were strongly and significantly associated with a decreased risk of obesity. Figure 1 shows the mean BMI of subjects with respect to categories of sports activity and TV/PC use in leisure time. The mean BMI in the groups with higher sports activity and less time spent for TV/PC is distinctly lower than in subjects who were not active in sports and spent a long time watching TV or using a PC during leisure time. In general, sports are mostly of moderate or vigorous intensity and are often executed in one bout without long interruptions, especially endurance activities like walking, running or cycling. These sports activities demanding high energy costs were most popular among active subjects in the present study. Even people of older age (≥ 65 years) were still active in endurance sports by being engaged in walking although PA was declining with rising age. In comparison, obese subjects are more likely to be engaged in activities of moderate intensity, but hardly perform activities of high intensity, such as many sports [28]. This contrasts to TV/PC use which is associated with a very low energy expenditure. With increasing sedentary behaviour physical activities decreases [29]; moreover, especially television watching is associated with snacking, leading to high caloric intakes [30].

Similar associations as reported here were found in other studies. An European study [31] investigating the PA pattern in samples from 15 EU member states found significant associations between BMI and leisure time PA [OR of 0.52 (0.43–0.64)] for subjects in the most active quintile compared to lowest and time spent sitting down [OR of 1.61 (1.33–1.95)] for subjects in the most inactive quintile compared to lowest, respectively. Cameron et al. [25] investigated the prevalence of obesity in Australian adults and examined its relationship with life-style factors. Strong associations between obesity and PA (OR of highest quintile: 0.70 men, 0.47 women) or TV watching (OR of highest quintile: 1.86 men, 1.82 women) were found. Similar associations between sedentary life-styles, mainly represented by TV watching, and PA have been shown by several previous studies [29,32-37].

In the present study, we could not find distinct associations between PA in activity domains other than sports and TV/PC use and the risk of obesity. In contrast to the results reported by King et al. [38], occupational PA was unrelated to obesity risk. For unemployed subjects the lowest though not significant point estimate was found; this finding is possibly due to the fact that students and those who retired or were unemployed had more time left for sports or other recreational activities. In several [29,40] but not all studies [39] an inverse association between occupational activity and leisure time PA was observed. The questioning for strenuous activities in leisure time mainly assessed moderate physical activities and contributed on average to only about 3 to 4 % of total daily energy expenditure. Risk estimates for obesity decreased with increasing activity in LTPA_strenuous _but did not reach statistical significance. This result may be affected by recall bias since obese subjects may have reported more activities in this category (Tab. 3) because of rating their activities more demanding.

Two studies reported an inverse association between sleep duration and obesity [29,41]. Except for the group with >8 MET*h/d spent with sleeping, in our study risk estimates of obesity decreased with increasing time spent with sleeping; however, results were not statistically significant. In the present study, also non-reported activities were not associated with obesity risk. Our questionnaire did not assess light-intensity activities of common life (e.g. eating, car driving, self-care, etc.). Consequently, the high proportion of time attributed to this PA domain – about half of estimated total daily energy expenditure – was almost expected. On the other hand this result supports the view that only a small part of daily energy expenditure is spent in demanding activities which should be remembered best [42].

The estimated level of total physical activity in terms of MET*h/d in the present study population was very similar to that reported in the NHAPS Study [43]. This study is one of the few assessing 24-h PA with computer-assisted telephone-interviews; they found in 7.515 subjects (aged 18 years and over) mean values of 39.9 kcal/kg for men and 37.8 kcal/kg for women (MET*h corresponds to kcal/kg). For comparison, mean values in our study were 40.56 MET*h/d and 38.47 MET*h/d among men and women, respectively (for medians see table 2). In a cohort of Swedish men aged 45–79 years, Norman et al. [9] reported a mean of 41.5 (SD: 4.9) MET*h/d for total daily activity assessed by questionnaire. In agreement with previous studies [10,31,32] total PA of the Bavarian subjects was found to be inversely associated with obesity. Subjects with a PAL value =1.75 (Q3 + Q4) had a 57 % reduced risk as compared to subjects with a PAL value <1.5. These findings fit with the WHO-recommendation that a PAL of 1.75 or more is necessary to avoid excessive weight gain, a recommendation which is based on the review of 40 international studies [2]. Among normal-weight subjects, 35.1% met the recommendation, which is still low but clearly higher than the 22.8 % in the obese subjects (table 5). Overall, this WHO-goal has only been reached by a total of 31.4 % of the study participants.

The public health-recommendation from the Centers for Disease Control and Prevention (CDC) and the American College of Sports Medicine (ASCM) of at least 30 minutes of moderate PA per day [4] was met by a total of 55.9 %. An identical rate was even achieved by obese subjects, which might be astonishing at first sight, but if the recommendation was considered only in terms of sports activities, the percentage of sufficiently active obese subjects dropped to only 20.7 %. Taking into account that the recommendation of 30 minutes of moderate PA per day has minimum-character in the context of weight-management yet remembering the stricter guidelines of 60 minutes stated by the Institute of Medicine (IOM) [5], the data would turn out even worse. Nevertheless, considering diverging methods of assessment and PA recommendations these results are quite comparable with other studies. Brown and Baumann [18] found that the subjects' percentage of meeting the current CDC/ACSM-recommendation in 2 Australian surveys ranged between 51.6 % and 60.2 %. Weyer et al. [44] observed that 61.5 % of 109 obese Germans did not meet any recommendation. This is less than the 87% of 7124 adults, who were not adequately active in the German General Health Survey in 1998 [45].

The obesity rate in this Bavarian sample is higher than in a recent survey published by the Federal Statistical Office of Germany [23] in 2004, but comparable to other German studies conducted since 1998. Bramlage et al. [24] reported on the prevalence of obesity comparing rates from the German "Hypertension and Diabetes Risk Screening and Awareness" (HYDRA)-study in 2001 (19.5 % in men, 20.3 % in women) with the German General Health Survey (GHS) 1998 data (18.8 % in men, 21.7 % in women). In comparison to the results of a former representative study in the Bavarian population in 1995 (BVS I), the prevalence of obesity increased in the last years as found also for other western countries [25-27].

The information about the participants' short-term PA was collected by means of three 24-hour telephone recalls, a method validated by Matthews et al. [14] (see methods section). Other methods like behavioural observation, use of motion sensors, physiological markers (e.g. heart rate) and calorimetry are less subject to bias in the assessment of mainly long-term PA and energy expenditure. Especially the double-labeled water method is regarded as 'gold standard'[15]. However, self-reported data obtained by means of diaries or recalls are most practical in large-scale population-based studies because of relatively low costs and low efforts for the participants [16]. In the present study, kind and duration of PA were assessed, but not the corresponding intensities (except for occupational PA). Instead, MET values were assigned to each specific activity. Consequently, some degree of error may have been introduced because of unclear description, misunderstanding or misidentification. In occupational PA, consideration of both self-reported job title and self-rated work-intensity at least reduced the great variability of subjects' individual performances within the same job title [17]. However, using mean MET values to express the intensity of a PA assumes that there are no individual differences in performing the same types of activities, an assumption which in practice does not hold true [7,8]. We further expressed PA in terms of MET*h/d and MET*h/24 h but avoided to express PA in terms of 'kcal' because the latter would have been strongly affected by body weight [7,8] thus resulting in misclassification of individuals [18]. Potential bias must also be considered due to typical problems of self-report. First, the BMI variable might be affected by overestimation of height and underestimation of weight [19,20] or in rare cases also by high muscle mass [21]. Using anthropometric measurements, valid BMI data could be obtained from a substantial part of the study subjects. Second, self-reported PA may be overestimated in order to create a more ideal picture of oneself [22]. And third, the quality of the survey is highly dependent on the respondents' memory, a source of bias that should be minimized due to the short recalling period of 24 hours [14]; this should be one of the major strengths of the current study, besides its representativeness and its relatively large sample size.

## Conclusion

The overwhelming part of the Bavarian population did not reach current PA recommendations, and subjects meeting the recommendations showed a significantly lower risk of obesity. Our results strengthen the view of promoting sports activity in expense to TV/PC use in leisure time in order to counterbalance the rising prevalence of obesity in the Bavarian population. Other PA domains like occupation, LTPA_strenuous_, sleeping and LTPA_non-reported _showed weaker or no associations with obesity risk. However, due to the cross-sectional study design, no conclusion on causality can be drawn. Especially for the PA category sports activity, it remains unclear whether people are obese due to the low PA or the low PA is a consequence of their high body fat content. With respect to the weight development over time, probably both views are correct.

## Competing interests

The author(s) declare that they have no competing interests.

## Authors' contributions

NS carried out the coding of activities and the statistical analysis, drafted the manuscript. HS participated in collection and processing of data, participated in the statistical analyses. SH participated in collection and processing of data. GK, KG, GW participated in fund raising and the design of the study, JL senior author responsible for the design of the study, participated in collection and analyses of data, drafted the manuscript. All authors read and approved the final manuscript.
